# Preparation and Characterization of Low-Molecular-Weight Natural Rubber Latex via Photodegradation Catalyzed by Nano TiO_2_

**DOI:** 10.3390/polym10111216

**Published:** 2018-11-01

**Authors:** Suhawati Ibrahim, Nadras Othman, Srimala Sreekantan, Kim Song Tan, Zairossani Mohd Nor, Hanafi Ismail

**Affiliations:** 1 School of Materials and Mineral Resources Engineering, Universiti Sains Malaysia, Engineering Campus, Nibong Tebal 14300, Penang, Malaysia; suhawati@lgm.gov.my (S.I.); srimala@usm.my (S.S.); ihanafi@usm.my (H.I.); 2 Technology and Engineering Division, Malaysian Rubber Board, Sungai Buloh 47000, Selangor, Malaysia; kstan@lgm.gov.my (K.S.T.); zairossani@lgm.gov.my (Z.M.N.)

**Keywords:** photodegradation, liquid natural rubber, UV light, TiO_2_ anatase, latex state

## Abstract

Natural rubber is one of the most important renewable biopolymers used in many applications due to its special properties that cannot be easily mimicked by synthetic polymers. To sustain the existence of natural rubber in industries, modifications have been made to its chemical structure from time to time in order to obtain new properties and to enable it to be employed in new applications. The chemical structure of natural rubber can be modified by exposure to ultraviolet light to reduce its molecular weight. Under controlled conditions, the natural rubber chains will be broken by photodegradation to yield low-molecular-weight natural rubber. The aim of this work was to obtain what is known as liquid natural rubber via photodegradation, with titanium dioxide nanocrystals as the catalyst. Titanium dioxide, which was firstly synthesized using the sol–gel method, was confirmed to be in the form of an anatase, with a size of about 10 nm. In this work, the photodegradation was carried out in latex state and yielded low-molecular-weight natural rubber latex of less than 10,000 g/mol. The presence of hydroxyl and carbonyl groups on the liquid natural rubber (LNR) chains was observed, resulting from the breaking of the chains. Scanning electron microscopy of the NR latex particles showed that titanium dioxide nanocrystals were embedded on the latex surface, but then detached during the degradation reaction.

## 1. Introduction

Natural rubber (NR), tapped from the *Hevea brasiliensis* tree, is the biggest bio-based polymer source at present [1]. Several million tons of NR are produced commercially each year as it is an important material in the production of tyres, gloves, liners, tubes, etc. NR currently ranks as the fourth most important natural resource after air, water, and petroleum [2]. As NR is a renewable resource, its applications should not be limited to common products, but can be diversified through modifications to the NR chains to produce new rubbers with new properties. The molecular structure of NR consists of a double bond in its repeating units of *cis*-1,4-isoprene, which can be modified with regard to specific functional groups such as epoxidized natural rubber (ENR) [3], acrylated NR [4], carbonyl telechelic natural rubber (CTNR) [5], and hydroxyl telechelic liquid natural rubber (HTNR) [6,7].

As a natural product, NR has a very high molecular weight, which has always been a hurdle during mixing. An intensive shearing process is needed to break down the molecular weight to a state wherein it is easier for the material to be blended with additives, shaped, and then vulcanized. Apparently, reducing the molecular weight of NR to obtain what is known as liquid natural rubber (LNR) has solved the mixing problem during compounding. LNR commonly has a molecular weight of less than 20,000 g/mol and is considered as a new material derived from NR. Furthermore, the degradation process also opens up the opportunity to use LNR in various applications such as in binders [8], adhesives [9], coatings [10], processing aids [11,12], compatibilizers [13], and encapsulation of paraffin wax [14], as well as precursors for new materials [15,16] and further modifications [17]. Consequently, reducing the molecular weight has extended the potential and possible applications of NR. Indeed, this old material can change the perception of its conventional use and open up new areas of applications, especially to challenge the properties of synthetic rubber [18]. Therefore, LNR is important for the sustainability of the NR industry, especially in expanding its potential to new areas of applications and new advanced materials. Currently, LNR can be produced by the mechanical, chemical, photochemical, sonication, or thermal degradation of NR, either in a dry, solution, or latex state.

In the photodegradation of NR, TiO_2_ has been determined to be an excellent catalyst in the presence of hydrogen peroxide to effectively oxidize the NR chain and scissor via the radical activity of the oxygen–oxygen single bond [19,20]. Hence, it has been shown that the LNR chain has formed hydroxyl and carbonyl groups at its extremities. LNR with a hydroxyl end group becomes attractive when its application can be expanded into new areas such as precursor specialty block polymerization [21,22], high-performance adhesives [9,23], and binders [8]. LNR has the same repeating unit (*cis*-1,4-isoprene) as virgin NR; consequently, they exhibit similar excellent characteristics such as flexibility, elasticity, low temperature, etc. 

Due to the increasing demand for a green and environmentally friendly process, the optimization of parameters and conditions for the photodegradation of NR in the latex state is being intensively explored [19,24,25]. Hence, the method of photodegradation developed by Ravindran [20] was duplicated and adopted for implementation in the latex state. Sakdapipanich et al. (2001) exposed NR latex to UV light in the presence of hydrogen peroxide, but no significant degradation was reported. This may have been due to the difficulty for the reagents and UV light to penetrate the membrane of the rubber particles [24]. In a recent work, when NR latex in the presence of hydrogen peroxide was exposed to UV light at an elevated temperature, significant degradation occurred wherein the molecular weight of the NR was reduced [25,26]. The use of TiO_2_ as a photocatalyst in the degradation of NR latex was also studied. TiO_2_ was assembled as a nanomatrix film to degrade NR latex under UV light in the presence of hydrogen peroxide, and it was reported to have yielded low-molecular-weight NR [19]. Even though the use of assembled nanomatrix TiO_2_ films yields a clean LNR, it has a limitation in that it is difficult to maximize the exposure of the film to UV light for optimum absorption and reaction. This problem can be overcome by adding TiO_2_ nanocrystals into the bulk latex, but the TiO_2_ would remain in the latex solution after the reaction has been completed. However, the remaining TiO_2_ nanocrystals in the LNR after the reaction can be useful either as a filler or catalyst for other advanced chemical reactions. TiO_2_ is well known as a good inorganic filler as it is chemically stable and biologically benign. The presence of TiO_2_ in NR has been found to increase the rate of stress relaxation [27], thermal conductivity, thermal diffusivity [28], UV protection [29], and antimicrobial activities [30] of the NR compound, in addition to its conventional role as an inorganic pigment in rubber products.

In this present work, the synergistic effect of the photodegradation of NR latex under UV light in the presence of hydrogen peroxide and TiO_2_ as a photocatalyst was investigated. TiO_2_ nanocrystals used in this study were prepared via the sol–gel method, and the effect of an acid catalyst and annealing temperature on the size and structure of the TiO_2_ was investigated. The anatase form of TiO_2_ was chosen as the photocatalyst in the degradation of NR latex. The reaction was carried out under a controlled temperature to obtain a degraded NR, which was then characterized for its structure and properties. The molecular weights of the NR and LNR were determined by means of gel permeation chromatography (GPC), whilst the molecular structure was determined using Fourier transform infrared (FTIR) spectroscopy and nuclear magnetic resonance (NMR) spectroscopy.

## 2. Materials and Methods

### 2.1. Materials

Titanium (IV) isopropoxide (TTIP), nitric acid (HNO_3_), and isopropanol were purchased from Sigma Aldrich (Saint Louis, MO, USA), and all these reagents were used without further purification.

The low-ammonia natural rubber latex (LATZ) used in this work was purchased from Lee Rubber Sdn Bhd (Selangor, Malaysia). Analytical-grade hydrogen peroxide was purchased from Merck (M) Sdn Bhd (Kenilworth, NJ, USA). Methanol, sodium dodecyl sulphate (SDS), and toluene were purchased from Fluka (Loughborough, UK). All the materials were used as received.

### 2.2. Preparation of TiO_2_ via Sol–Gel Method

Solutions of TTIP in isopropanol (solution A) and water in isopropanol (solution B) were prepared. Under vigorous stirring, solution A was added dropwise into solution B. The molar ratio of water/TTIP was fixed at 115, and the pH of solution B was varied from 1 to 7 using HNO_3_. The sol obtained was washed thrice and dried overnight in an oven at 60 °C. The TiO_2_ was then ground in a mortar and was calcined in a furnace at temperatures between 300 to 700 °C for 2 h.

### 2.3. Photodegradation of NR Latex

LATZ latex was mixed with 2 phr of SDS, and the required amount of TiO_2_. Water was then added to obtain a dry rubber content (DRC) of 10%. The solution was then poured into a reaction flask and was heated to 65 °C before hydrogen peroxide was dropped into the solution. The parameters and temperature used were similar to those used in our previous work [25]. Next, the solution was left under a 30 watt UV light (365 nm) for 48 h. The degraded latex was then coagulated using methanol and washed a few times with water, after which it was dried in a vacuum oven. The purification of the sample was carried out by dissolving the yield in toluene and re-precipitating it in methanol, followed by drying in a vacuum oven until a constant weight was achieved.

### 2.4. Characterizations

X-ray diffraction (XRD) was used to study the crystallization of TiO_2_. The measurement was performed using a Bruker D8 Advance diffractometer (Karlsruhe, Germany) operating in the reflection mode with Cu Kα radiation (35 kV, 30 mA) and a diffracted beam monochromator using a step scan mode with a step of 0.075° (2θ) at 4 s per step. The diffraction patterns of both the anatase and rutile TiO_2_ powders were compared with reference to the Join Committee on Powder Diffraction Standards (JCPDS) database.

Transmission electron microscopy (TEM, FEI, Eindhoven, The Netherlands) was used to study the morphology of TiO_2_. A sample of the diluted latex containing TiO_2_ nanocrystals was first sonicated in an ultrasound bath for 1 h, followed by immediate transfer to a copper grid using a pipette. The copper grid was allowed to dry completely at ambient temperature before being inserted into the TEM, an FEI Tecnai G20. The TEM images were taken at 200 kV at various magnifications. High-resolution TEM (HRTEM) images for the nano TiO_2_ where fringes of the crystal could be clearly observed were also recorded using the same instrument. For the LNR, the sample was diluted with toluene, drop cast onto a copper grid, and allowed to dry under ambient temperature before being inserted into the transmission electron microscope.

Viscotek multi-detector gel permeation chromatography (GPC, Malvern, UK) was used to measure the weight-average molecular weight (Mw) and the number-average molecular weight (Mn) of the prepared LNR samples. The measurements were carried out at 30 °C using tetrahydrofuran (THF) as the mobile phase and polyisoprene as the internal standard.

Crosslinking of the sample was studied in terms of the mean gel content. The gel content was determined by dissolving about 2 g of the sample (m_0_) in 100 mL of toluene and keeping it for one week in a dark place. The gel fraction was then filtered and dried (m_1_) in an oven at 60 °C for 24 h. The percentage ratio of gel and the original sample was estimated to be the gel content, as shown in Equation (1).

(1) $$\mathrm{Gel}\;\mathrm{content}\;\left(\%\right)=\frac{\mathrm{Weight}\;\mathrm{of}\;\mathrm{dried}\;\mathrm{gel}\left(m_{1}\right)}{\mathrm{Weight}\;\mathrm{of}\;\mathrm{sample}\;\left(m_{0}\right)}\times 100$$

A Fourier transform infrared (FTIR) spectroscopy analysis was done using a Thermo Nicolet 6700 FTIR spectrometer (Waltham, MA, USA), and the Diamond Attenuated Total Reflectance (DATR) technique was used for the analysis. The sample was analyzed in the transmittance mode within the range of 4000–600 cm^−1^ at a resolution of 4 cm^−1^ and 64 scans per sample.

The ^1^H and ^13^C NMR spectra were recorded for a sample that was dissolved in deuterated chloroform and was analyzed using a Bruker 500 spectrometer (Billerica, MA, USA) with TMS as the internal standard at room temperature. The ^1^H NMR spectrum was measured with 1024 scans at a frequency of 500 MHz, while the ^13^C spectrum was measured with 16,384 scans at a frequency of 125 MHz and spinning rate of 20 Hz.

The morphology of the natural rubber latex surface was taken on a JOEL FE-SEM JSM 6701F (Tokyo, Japan) that was operated at 2.0 kV. Prior to the observation, the sample was placed onto a specimen stub and was sputter-coated with an ultra-thin layer of platinum, which was approximately 100 Å thick, to reduce charging.

## 3. Results and Discussion

### 3.1. Preparation and Characterization of TiO_2_ Nanocrystals

TiO_2_ synthesized via the sol–gel method will undergo two simultaneous reactions (hydrolysis and condensation) when organometallic precursors react with water. These two reactions are sensitive to many experimental parameters such as raw material concentration, pH, hydrolysis temperature, mixing conditions, annealing time, and annealing temperature [31,32].

#### 3.1.1. Effect of pH

**Figure 1:**
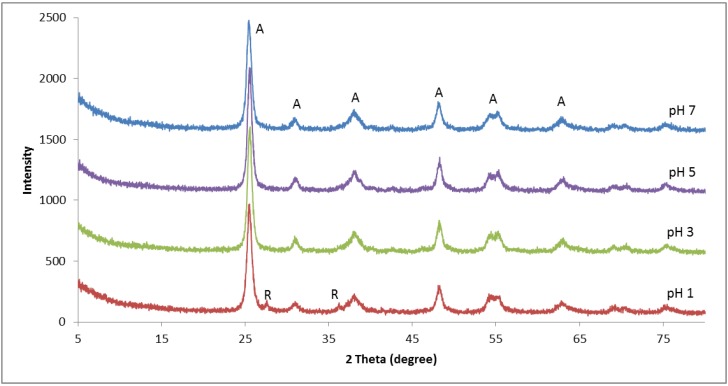
XRD patterns of TiO_2_ prepared at various pH, where A and R denote the XRD peaks assigned to the anatase and rutile phases of TiO_2_, respectively.

Figure 1 shows the XRD patterns of the TiO_2_ powders prepared at pH 1 to 7 and subsequently annealed at 500 °C for 2 h. The samples that were prepared at pH 3, 5, and 7 exhibit peaks at 2θ of 25°, 30°, 37°, 47°, 53°, 55°, and 62°, corresponding to the (101), (121), (004), (200), (105), (211), and (204) planes of the TiO_2_ anatase phase. However, for the sample prepared at pH 1, the anatase peaks appeared together with small peaks at 2θ of 27°, 36°, and 56°, indicating the presence of a rutile phase. Nevertheless, these results contradict the results reported by Nolan et al. [33], where it was claimed that reducing the acidity of the sol had resulted in a lowering of the anatase to a rutile transformation temperature. However, this contradiction may be due to differences in the preparation procedures and materials used. 

Apparently, the crystallization of TiO_2_ is affected by parameters of the treatment process such as the annealing and the atmosphere of the reaction [34,35]. Furthermore, parameters such as the pH of the media, the solvent, precursor concentration, annealing temperature, and annealing time will influence the properties of the TiO_2_ obtained. Consequently, the presence of an acid as a hydrolysis catalyst influences both the condensation rate and the structure of the TiO_2_ [36].

In a sol–gel synthesis, two simultaneous reactions (hydrolysis and condensation) take place when titanium alkoxides are hydrolyzed and subsequently polymerized to form a three-dimensional oxide network, represented schematically as follows:

Ti(OR)_4_ + 4H_2_O → Ti(OH)_4_ + 4ROH (hydrolysis) (2)

Ti(OH)_4_ → TiO_2_·nH_2_O + (2 − n)H_2_O (condensation) (3)

where R is ethyl, i-propyl, n-butyl, etc. [32]. Due to the high acidity of the tetravalent cation, an unstable hydroxide Ti(OH)_4_ is formed. Oxolation and olation will then proceed simultaneously during nucleation to form an amorphous oxide, TiO_2_.nH_2_O. The presence of the H^+^ ion will catalyze the hydrolysis by protonating the hydroxide group, which then prevents agglomeration from occurring to cause the formation of gel. Generally, inorganic acids such as HCl, HNO_3_, and H_2_SO_4_ are used as acid catalysts. Thus, a low (acidic) pH promotes the formation of an anatase structure, whilst an amorphous structure is obtained at a high (alkaline) pH [32].

**Figure 2:**
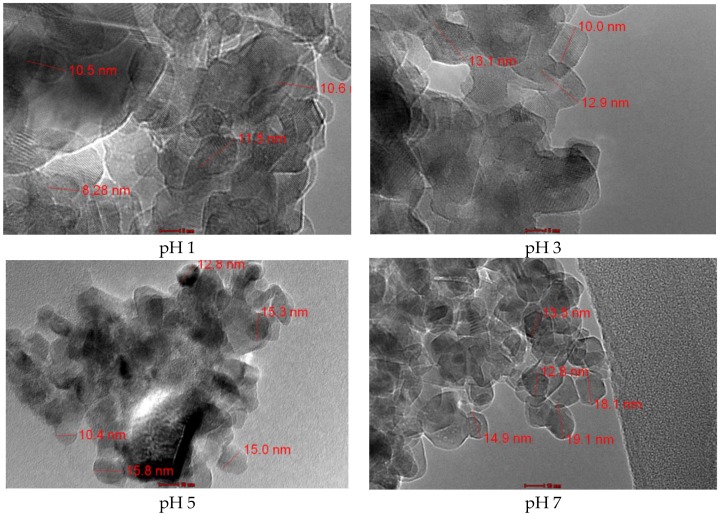
TEM micrographs of TiO_2_ nanocrystals prepared by a sol–gel process at pH 1 to 7.

Figure 2 shows the TEM micrographs of the TiO_2_ nanocrystals prepared at pH 1, 3, 5, and 7. The average sizes were 10.2, 12.0, 13.9, and 15.7 nm for the TiO_2_ synthesized at pH 1, pH 3, pH 5, and pH 7, respectively. The size of the TiO_2_ nanocrystals prepared at a lower pH was found to be smaller than those prepared at a higher pH. In an acidic medium, the rate of particle formation is slower compared to in neutral and alkaline media [37], especially when the hydrolysis reaction is catalyzed by the presence of protons through protonation of the leaving group [38,39]. In the meantime, the protonation of the OH group in the Ti cluster inhibited nucleophilic attacks on the other clusters that prevented precipitation and led to gelation [38]. Hence, the TiO_2_ particles were reported to be smaller in size compared with the particles in the neutral and alkaline media.

#### 3.1.2. Effect of Annealing Temperature

**Figure 3:**
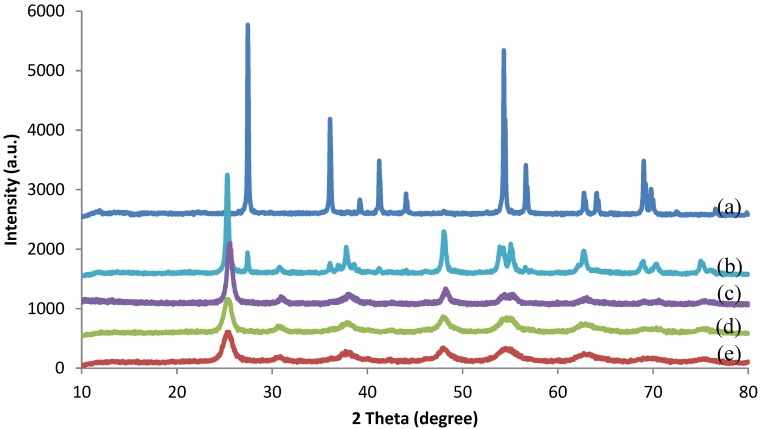
XRD patterns of TiO_2_ nanocrystals annealed at various temperatures: (**a**) 700 °C, (**b**) 600 °C, (**c**) 500 °C, (**d**) 400 °C, (**e**) 300 °C.

Figure 3 shows the XRD pattern of the TiO_2_ nanocrystals prepared at pH 3 and subsequently annealed at 300 to 700 °C. The TiO_2_ nanocrystals annealed at 300, 400, and 500 °C exhibited peaks at 2θ of 25°, 30°, 37°, 47°, 53°, 55°, and 62° corresponding to the (101), (121), (004), (200), (105), (211), and (204) planes of the anatase phase (Figure 3a–c). However, when the annealing temperature was increased to 600 °C, the rutile peaks at 2θ of 27°, 36°, and 56° appeared (Figure 3d). Beyond 700 °C, the anatase phase was completely transformed to the rutile phase (Figure 3e). During the annealing process, the metastable anatase phase was transformed into a thermally stable rutile phase, which was in agreement with another reported work [40].

Among the three polymorphs of TiO_2_, the anatase form has been widely used as a popular catalyst due to its various merits, such as optical and electronic properties, high photocatalytic activity, low cost, nontoxicity, and chemical stability [41,42,43], and it is being widely used in the degradation of organic pollutants [44,45]. Therefore, in this work, the TiO_2_ that was prepared in pH 3 and annealed at a temperature of 500 °C for 2 h was used as a catalyst in the photodegradation of NR latex.

### 3.2. Photodegradation of NR Latex

The degradation of NR in the presence of TiO_2_ is similar to the decomposition of most other organic materials. The activation of TiO_2_ under UV light involves the migration of photon-generated electrons (*e−*) and holes (*h+*) to the surface to serve as redox sources, which then attack the organic material. Furthermore, the use of nano-sized TiO_2_ increases the photocatalytic activities toward the latex particles due to higher coalescence with a bigger surface area of TiO_2_, by which lower-molecular-weight LNR can be obtained. Among the TiO_2_ polymorphs, the anatase structure shows the highest photocatalytic activities possessed by the larger band gap and better surface properties [41].

#### 3.2.1. Effect of TiO_2_ Nanocrystals on Photodegradation

Table 1 shows the molecular weights and gel contents of LNRs that were prepared with various amounts of TiO_2_ nanocrystals. The molecular weight of NR was found to have been reduced to 18.6 × 10^3^ g/mol after photodegradation without the addition of TiO_2_ nanocrystals. In the presence of TiO_2_ nanocrystals, the molecular weight was further reduced to 15.8 × 10^3^ and 7.3 × 10^3^ g/mol when 0.4 and 0.8 phr, respectively, of TiO_2_ nanocrystals were added. However, when the amount of TiO_2_ nanocrystals was increased to more than 0.8 phr, the molecular weight of the LNR was found to increase together with the polydispersity value. The increase was expected due to the presence of a high amount of radical species, which induced side products such as crosslinking and chain reconnecting, as indicated by the increase in the polydispersity and gel content [20,46,47]. This would explain the increase in the gel content and polydispersity of the degraded rubber when the amount of TiO_2_ was increased even when the molecular weight decreased. A high amount of TiO_2_ nanocrystals would have increased the kinetics of reactions rapidly; hence, more side reactions such as crosslinking and chain connecting occurred. These side reactions would have increased and become uncontrolled due to the simultaneous effect of a high concentration of TiO_2_ during exposure to UV light [48].

#### 3.2.2. FTIR Analysis of LNRs

**Figure 4:**
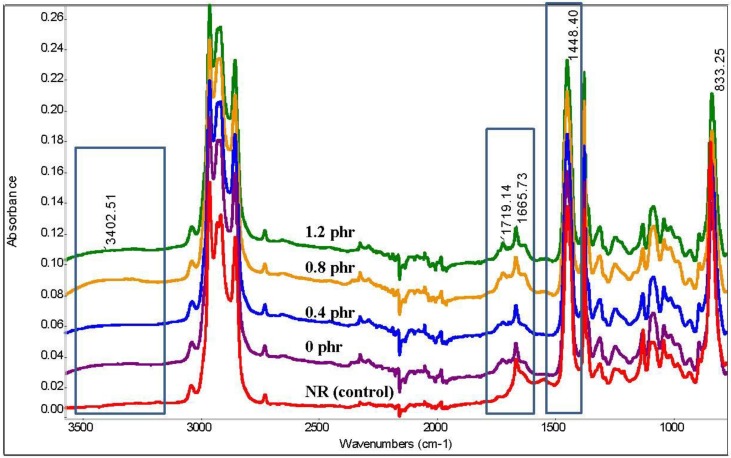
FTIR spectra of NR and LNRs prepared with various amounts of TiO_2_.

Figure 4 shows the FTIR spectra of the NR and LNRs that were prepared with various amounts of TiO_2_ nanocrystals ranging from 0 to 1.2 phr. Generally, the spectra of the LNRs showed a similar pattern to that of the NR spectrum. However, there was a depletion in the peaks, indicating the breaking of chains, and an increase in some peaks, indicating the presence of terminal groups as a result of the chains breaking.

Table 2 shows the intensity of the IR changes in the respective peaks for the NR and LNRs prepared with and without TiO_2_. The calculated values were solely based on the peak areas—as it was difficult to find a reference peak to make a comparison—and the ratio. The peaks at 1660 and 1445 cm^−1^ that correspond to the C=C and C–C bonds are commonly used as a reference, but these have been found to change after degradation. The discussions below refer to the respective peak changes as tabulated in Table 2.

The peak areas of 1660 and 1445 cm^−1^, which correspond to the C=C and C–C bonds, decreased after degradation for all the LNR samples. Both groups were expected to be attacked, and hence, the NR chains were broken during degradation. The depletion of the C=C and C–C bonds suggests that both groups were involved in the chain breaking by means of at least two different mechanisms, namely, oxidation at the C=C bond by the oxygen species and the activity of radicals at the C–C bond. Furthermore, both groups were also able to absorb UV light to enter into an excited state, followed by chain breaking [49,50,51].

The broad peak at 3400 cm^−1^, which corresponded to the vibrations of the bonded OH group, was increased in the spectra of the LNRs, thereby indicating the presence of OH groups on the LNR chains. Among the samples, the LNR prepared with 0.8 phr of TiO_2_ showed the highest peak intensity in this region, thereby indicating the existence of the highest number of OH groups on the LNR chain. A similar pattern was also observed for the weak peak at 1720 cm^−1^ which corresponded to the carbonyl group. As discussed above, this LNR sample showed the lowest molecular weight; hence, a greater number of terminal end groups should have been observed, as indicated in the spectrum. Hydroxyl and carbonyl groups are commonly found in degraded polymers as a result of chain breaking during photodegradation [52,53].

Hydrogen peroxide_,_ on exposure to UV light, decomposes to a hydroxyl radical that is a very reactive species. Its concentration will be higher when this radical is generated in an illumination process, where TiO_2_ has been activated after absorbing photons from UV light [54,55,56], and this relates to the surface area of TiO_2_ [43]. This radical is capable of breaking the NR chains by attacking the C–C bond and leaving the hydroxyl as a terminal group [46]. Besides this, hydrogen peroxide is also well known as a strong oxidation agent that is able to oxidize and break the NR chain at the C=C bond, leaving the carbonyl group as a terminal end group [57,58]. At the same time, the C=C bond absorbs UV light to cause photooxidative degradation and break the NR chain, thereby also leaving the carbonyl group as a terminal end group [48]. The presence of nano-sized TiO_2_ certainly increases the formation of OH radicals due to a higher surface area.

**Figure 5:**
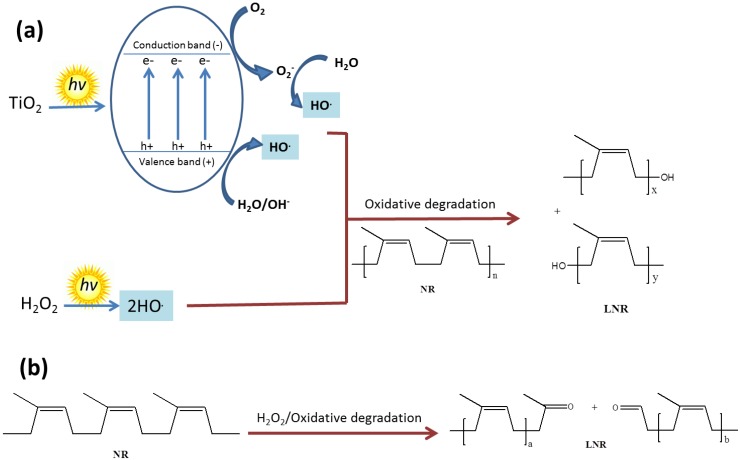
Diagram of oxidative degradation of NR under UV light in the presence of TiO_2_ by (**a**) a hydroxyl radical and (**b**) hydrogen peroxide as an oxidizing agent.

Figure 5 illustrates the mechanisms that can possibly occur during the degradation of NR under UV light in the presence of TiO_2_ as a photocatalyst. The generation of hydroxyl radicals, either from the activities of TiO_2_ in an excited state or upon decomposition of hydrogen peroxide under UV light, is depicted in Figure 5a. It is possible that this reactive species attacks the C–C bond of NR, whereby it can easily break up under favorable conditions such as in the presence of free radicals. The protons that are bonded to this alpha carbon (C_α_H_2_–C_α_H_2_) are labile protons due to the *cis* configuration of the isoprenic units. These groups are not in the same plane and provide an unbalanced structure with the pendent methyl groups, resulting in stearic hindrance. In the presence of hydroxyl radicals, the labile protons will be abstracted and inserted into the hydroxyl groups during the chain-breaking process [59,60]. The broken C=C bonds in the NR chains can be oxidized by hydrogen peroxide, which is a strong oxidizing agent, to leave a carbonyl as the end group, as shown in Figure 5b.

#### 3.2.3. NMR Analysis of LNRs

**Figure 6:**
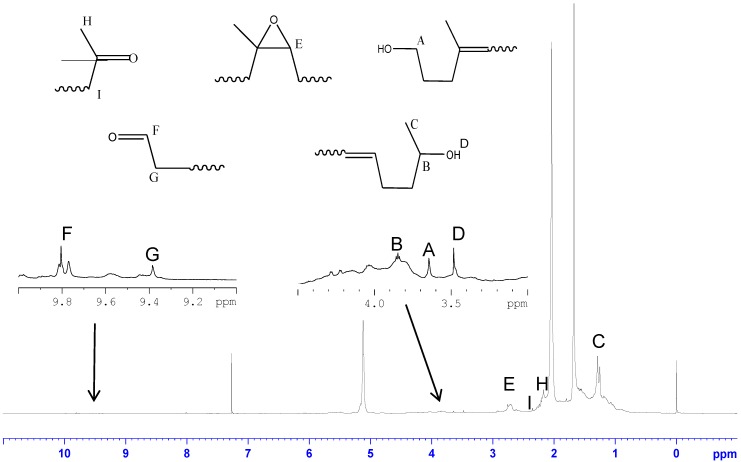
^1^H NMR spectrum of LNR prepared with 0.8 phr TiO_2_.

Figure 6 shows the ^1^H NMR spectrum of LNR which was prepared with 0.8 phr TiO_2_ nanocrystals. Typical NR peaks were assigned to the chemical shifts (ppm) 1.68 (–C**H**_3_), 2.05 (–C**H**_2_), and 5.13 (=C**H**–) [61]. The small peaks at 3–4 ppm indicated the presence of hydroxyl groups on the LNR chains at different locations. These new small signals at the chemical shifts of 1.28, 3.47, 3.65, and 3.84 ppm could be assigned to methylene protons adjacent to the secondary alcohol, protons of the hydroxyl group, hydroxylated methane protons, and hydroxylated methylene protons, respectively [62,63]. The pattern of this spectrum was similar to that in our previous work for LNR prepared without TiO_2_ [26].

The presence of carbonyl groups on the LNR chain was also observed for both aldehyde and ketone groups. The aldehyde group was indicated by the presence of small peaks at the chemical shift of 9.8 ppm for aldehyde protons and 9.38 ppm for α,β-unsaturated aldehyde protons, while the ketone group was detected at the chemical shift of 2.14 ppm for ketone methylic protons and 2.25–2.48 ppm for α,β-unsaturated ketone protons. Since the reaction was carried out under atmospheric air and in latex, reactions involving oxygen could not be avoided and occurred as side reactions. The oxidation reactions by singlet oxygen, radical-induced auto-oxidation from hydrogen peroxide, and hydroperoxide photolysis resulting from UV light led to the formation of by-products such as hydroperoxy, hydroxyl, carbonyl, and epoxide groups [63]. These results were in agreement with reports by previous researchers that the degradation of NR by an oxidizing agent and hydroxyl radical leaves carbonyl and hydroxyl groups as the end groups [26,57,64,65,66].

**Figure 7:**
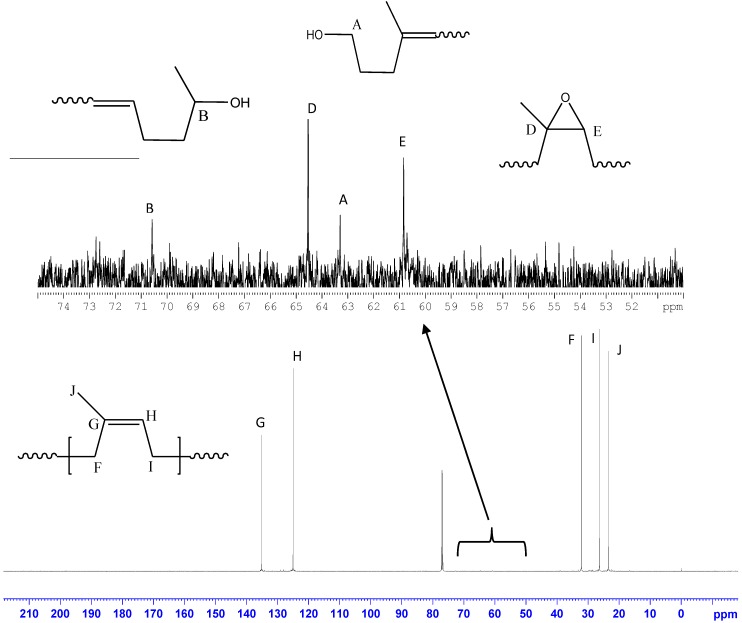
^13^C NMR spectrum of LNR that was prepared with 0.8 phr TiO_2_.

Figure 7 shows the LNR spectrum of ^13^C NMR to confirm its structure and the reactive groups present on its chain. The spectrum indicates the presence of typical peaks of NR at chemical shifts of δ 23.51 ppm (–C(CH_3_)=CH–), 26.44 ppm (–C(CH_3_)=CHCH_2_), 32.12 ppm (–CH_2_C(CH_3_)=CH–), 124.98 ppm (–C(CH_3_)=CH–), and 135.16 ppm (–C(CH_3_)=CH–). Besides this, small peaks corresponding to terminal groups were also observed at chemical shifts of δ 63.30 and 70.60, which were assigned to hydroxylated methylene and tertiary carbon groups, respectively, thereby confirming the presence of the hydroxyl end group. Further, signals were also observed at chemical shifts of δ 60.78 and 64.58 ppm, which were assigned to carbons of the epoxy group. The epoxy groups would come from those naturally existing in NR chains [67] and also from a side reaction of photodegradation [63].

The determination of reactive groups such as hydroxyl and carbonyl groups on the LNR chain is quite challenging as the quantity of these groups is extremely low compared to the whole mass of LNR. In this present work, ^1^H and ^13^C NMR showed a reliable signal-to-noise ratio and good resolution to determine and identify the presence of end groups which resulted from chain-breaking during the photodegradation reaction [16].

#### 3.2.4. SEM and TEM Micrographs of Latex Particles

**Figure 8:**
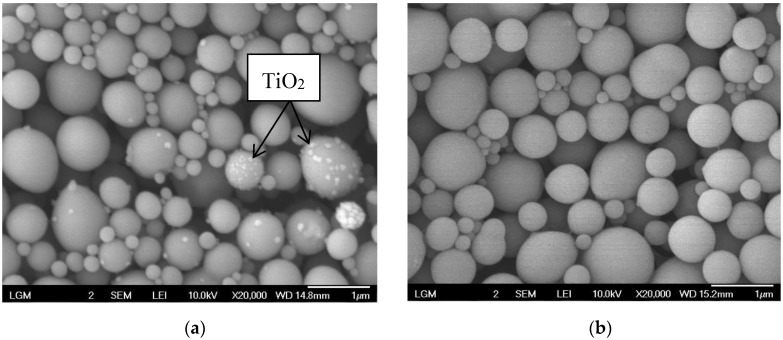
SEM micrographs of NR latex particles in the presence of TiO_2_ (**a**) before reaction (20 K magnification and (**b**) after reaction (20 K magnification).

The SEM micrographs of NR latex particles before and after photodegradation are shown in Figure 8a,b, respectively. From the micrographs, it is evident that the latex particles were able to maintain their shape, and no destruction was observed after the photodegradation. Before the photodegradation, TiO_2_ nanoparticles could be clearly observed to be embedded on the NR latex particles. The existence of the TiO_2_ nanoparticles could be clearly seen in the clusters, which had a size of more than 100 nm. However, the TiO_2_ nanoparticles were not dispersed homogeneously on the surface of the particles. Figure 8a shows that the TiO_2_ nanoparticles were embedded on certain latex particles only, depending on the introduction of the TiO_2_ nanoparticles to the latexes. However, TiO_2_ nanoparticles were not observed on the surface of the latex particles after photodegradation, as depicted in Figure 8b. Further analysis using TEM was carried out to confirm the position of TiO_2_ on the surface of latex particles. 

**Figure 9:**
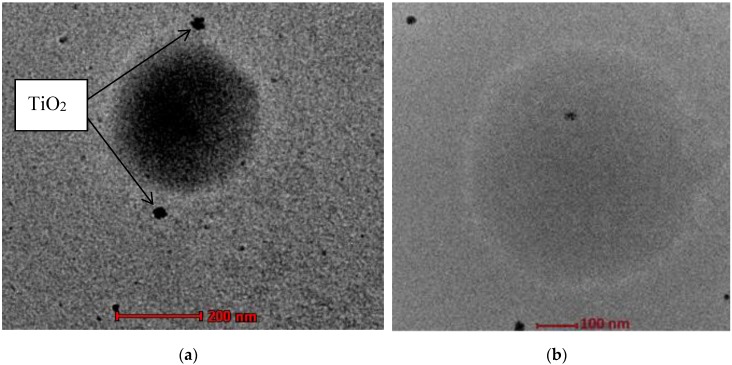
TEM micrographs of NR latex particles in the presence of TiO_2_ (**a**) before and (**b**) after the reaction.

Figure 9 shows the TEM micrographs of NR latex particles before (Figure 9a) and after (Figure 9b) photodegradation. The micrograph of the NR latex particles before photodegradation shows that the TiO_2_ nanoparticles were located at the outer layer of the latex surface, which was the non-rubber layer of the latex particles. The whiter layer around the latex particles is referred to as the non-rubber layer [68]. Even though the size of the TiO_2_ nanoparticles used was about 10 to 15 nm, they were embedded onto the latex surface as aggregates with sizes of about 100 to 140 nm. These aggregates could not penetrate into the latex membrane but instead were embedded on the outer layer or the non-rubber layer only. The TEM result for the sample after photodegradation supported the micrograph obtained from the SEM, where the TiO_2_ nanocrystals were not observed on the surface of the LNR latex particles. The desorption of the TiO_2_ nanocrystals from the latex surface during photodegradation may have been due to some changes in the composition of the non-rubber layer during the reaction. A further investigation is required to confirm the hypothesis, but it will not be discussed in this paper.

## 4. Conclusions

Functionalized LNR was successfully prepared via photodegradation catalyzed by TiO_2_ anatase nanocrystals. TiO_2_ as a photocatalyst was prepared via the sol–gel method. The presence of TiO_2_ increased the efficiency of the photodegradation of NR. The molecular weight of NR was significantly reduced from 549.3 × 10^3^ g/mol to 7.3 × 10^3^ g/mol when 0.8 phr of TiO_2_ nanocrystals was used. The LNR obtained was observed to have depleted FTIR signals corresponding to the C–C and C=C bonds compared to the virgin NR, indicating that chain-breaking had occurred in both these groups during the degradation reaction. These sites might have been attacked either by hydroxyl radicals or oxygen species formed from the photocatalytic activities of TiO_2_ and hydrogen peroxide under UV light. The broken chains then formed hydroxyl and carbonyl groups at the terminals, where these groups were found to have increased on the LNR chain.

## Figures and Tables

**Table 1:** Molecular weights and gel contents of liquid natural rubbers (LNRs) prepared with and without TiO_2_.

TiO_2_ (phr)	Mn (×10^3^) (g/mol)	Standard Deviation of Mn	Polydispersity	Gel Content (%)	Standard Deviation of Gel Content
NR	549.3	3.20	7.5	19.2	1.69
0	18.6	1.09	5.00	0.09	3.53
0.2	19.1	0.88	3.01	0.24	0.11
0.4	15.8	1.35	3.60	0.49	0.60
0.8	7.3	2.34	8.00	0.57	0.48
1.2	12.7	0.16	6.08	0.69	0.27
1.6	20.1	0.86	5.19	0.93	0.18

**Table 2:** Peak areas of IR spectra for NR and LNRs that were prepared with various amount of TiO_2_.

Peaks (cm^−1)^	Peak Assignment	Peak Area				
		NR	Amount of TiO_2_ (phr)			
			0	0.4	0.8	1.2
3400	C-OH stretching	0.211	0.960	0.694	1.699	0.865
1720	Carbonyl	0.002	0.085	0.056	0.114	0.119
1661	C=C stretching	0.809	0.388	0.325	0.490	0.455
1445	CH_2_ bending	3.712	3.446	3.493	3.397	3.455
